# Gender and the Grandfather Caregiver Experience

**DOI:** 10.1093/geroni/igaa057.1646

**Published:** 2020-12-16

**Authors:** AviElle Raymore

**Affiliations:** West Virginia University, Point Pleasant, West Virginia, United States

## Abstract

In the United States, 2.7 million grandparents are responsible for a grandchild in their home. Grandfathers are present in the majority of grandparent caregiver households, but their contributions and voices are often overlooked. The aim of this study was to explore how grandfathers experience caregiving as men. Twelve grandfathers from the age of 50-76 years participated in the study. Two face-to-face, semi-structured interviews were conducted with eleven grandfather caregivers while a telephone interview was conducted with one grandfather. Interviews focused on their life story, experiences as grandfather caregivers, and views on male caregiving. Data were analyzed using coding and thematic analysis. Gender was important throughout grandfather’s caregiving experiences. Grandfathers discussed their attitudes towards caregiving using language that reflected traditional gender norms. To them, women were nurturing caregivers while men were supposed to provide for their families as caregivers. Grandfathers appeared to stay connected to notions of traditional masculinities through participation in sports and physical play with their grandchildren and through their emphasis on men as responsible and providers. Grandfathers were aware that others may view them as incompetent caregivers, but they did not allow these stereotypes to affect how they viewed themselves as caregivers. These findings can improve the understanding of this population for service providers who work with grandparent caregivers. Providing better outreach for grandfather caregivers, strengthening programs and supports for them, and confronting attitudes or views towards male caregiving are important practice implications.

